# Association of Dietary Patterns with Components of Metabolic Syndrome and Inflammation among Middle-Aged and Older Adults with Metabolic Syndrome in Taiwan

**DOI:** 10.3390/nu10020143

**Published:** 2018-01-28

**Authors:** Ahmad Syauqy, Chien-Yeh Hsu, Hsiao-Hsien Rau, Jane C.-J. Chao

**Affiliations:** 1 School of Nutrition and Health Sciences, College of Nutrition, Taipei Medical University, 250 Wu-Hsing Street, Taipei 11031, Taiwan; syauqy@fk.undip.ac.id; 2 Department of Nutrition Science, Faculty of Medicine, Diponegoro University, Jl. Prof. H. Soedarto, SH., Tembalang, Semarang City, Central Java 50275, Indonesia; 3 Department of Information Management, National Taipei University of Nursing and Health Sciences, 365 Ming-Te Road, Peitou District, Taipei 11031, Taiwan; cyhsu@ntunhs.edu.tw; 4 Master Program in Global Health and Development, College of Public Health, Taipei Medical University, 250 Wu-Hsing Street, Taipei 11031, Taiwan; 5 Graduate Institute of Biomedical Informatics, College of Medical Technology, Taipei Medical University, 250 Wu-Hsing Street, Taipei 11031, Taiwan; rauhhh@gmail.com; 6 Nutrition Research Center, Taipei Medical University Hospital, 252 Wu-Hsing Street, Taipei 11031, Taiwan

**Keywords:** dietary patterns, metabolic syndrome, inflammation, Taiwan

## Abstract

This study examined the correlation of dietary patterns with components of metabolic syndrome (MetS) and inflammation among middle-aged and older adults with MetS in Taiwan. This cross-sectional study used data from the Mei Jau International Health Management Institution in Taiwan between 2004 and 2013. A total of 26,016 subjects aged 35 years and above were selected for analysis. MetS was defined according to the International Diabetes Federation. Three dietary patterns were identified by principal component analysis. High intake of a meat–instant food dietary pattern (rich in animal protein, saturated fat, sweets, sodium, and food additives) was positively associated with components of MetS and C-reactive protein (CRP), while high intake of a vege–seafood dietary pattern (rich in dietary fiber, vitamins, minerals, and unsaturated fat) or a cereal–dairy dietary pattern (rich in dietary fiber, antioxidants, phytochemicals, complex carbohydrate, prebiotics, and probiotics) was inversely associated with components of MetS and CRP. Our findings suggested that intake of a vege–seafood dietary pattern or a cereal–dairy dietary pattern decreased the risk of developing MetS and inflammation among middle-aged and older adults with MetS.

## 1. Introduction

The International Diabetes Federation (IDF) defines metabolic syndrome (MetS) as a cluster of conditions characterized by central obesity with two of the following four factors: elevated blood pressure, reduced high-density lipoprotein-cholesterol (HDL-C), elevated blood serum triacylglycerol (TG), and elevated fasting plasma glucose (FPG). Central obesity is considered to be the underlying cause of MetS, as it is associated with insulin resistance and contributes to MetS and its components [1]. MetS has been considered as a significant determinant of cardiovascular diseases (CVDs). Recently, CVD—a major public health concern throughout the world—contributed to 30% of deaths worldwide [2]. People with MetS are also more likely to have inflammation that could potentially aggravate the incidence of CVD [3,4]. Furthermore, the prevalence of MetS has significantly increased in all regions around the world [5,6,7,8,9,10]. A previous study conducted in China found that the prevalence of MetS was 24.5% in 2016 [11]; in Taiwan, a study using the Nutrition and Health Survey in Taiwan (NAHSIT) showed an increasing trend of MetS from 13.6% to 25.5% from NAHSIT 1993–1996 to NAHSIT 2005–2008, respectively [12]. The prevalence of central obesity also increased with age in Taiwan. The prevalence of central obesity in Taiwanese people aged from 30 to 39 years old was 27.0% in men and 16.4% in women; furthermore, the prevalence increased considerably in Taiwanese people aged from 70 to 79 years old, 42.9% and 64.6% for men and women, respectively [13].

Many factors such as demographics and lifestyle are known to be involved in the etiology of MetS [14]. Among these factors, dietary patterns have demonstrated an important role in the increased prevalence of MetS [15,16]. When evaluating the association of diet and disease, dietary patterns consisting of complex foods with many nutrients are more prominent influencing factors than single nutrients or diet items. Hence, holistic dietary patterns are considered to be better at preventing the development of diseases than single foods [17]. Healthy dietary patterns such as the traditional unrefined diet and the Mediterranean diet (which are both characterized as rich in vegetables, fruit, whole grains, cereal, fish and seafood products), as well as the low-fat diet, showed positive association with the components of MetS. A previous study found that a diet containing more fruit, vegetables, and whole grains decreased MetS and inflammation [18]. However, unhealthy dietary patterns including the Western dietary pattern—characterized as rich in red meat, organ meat, processed foods, and high-fat foods—was negatively associated with MetS [19,20,21,22]. Moreover, the Western dietary pattern might increase the risk of MetS and promote the production of pro-inflammatory markers [23].

Some studies have investigated the correlation between dietary patterns and diseases. Previous studies showed a significant correlation of dietary patterns with CVD [24] and cognitive function [25] in Taiwanese middle-aged and/or older adults. Additionally, people who ate more fruit and vegetables and less animal-derived foods had a better quality of life and less total health care costs in Taiwan [26]. A review study on the metabolic health effect of dietary habits among middle-aged and older adults has been discussed [27]. However, no study has been found that clarifies the association of dietary patterns with MetS and inflammation in Taiwan. The objective of this study was to investigate the correlation of dietary patterns with components of MetS and inflammation among middle-aged and older adults with MetS in Taiwan.

## 2. Materials and Methods

### 2.1. Study Design

This study was performed using a cross-sectional design to analyze data from the Mei Jau (MJ) International Health Management Institution in Taiwan between 2004 and 2013. The MJ International Health Management Institution is an independent health screening and management institution in Taiwan. It has four screening centers (Taipei city, Taoyuan city, Taichung city, and Kaohsiung city). They offer comprehensive health screenings for individuals who seek health check-ups. All participants who came to the MJ screening centers were from the general population. When visiting the screening centers, prior to undergoing a health examination, the participants of the study agreed to answer a structured questionnaire and signed a consent form, which allowed the MJ International Health Management Institution to collect the data for research use only without their personal information. All data were collected by MJ health screening centers. The health screening centers have been defined clearly in previous studies [24,28]. This study was approved by the Taiwan Medical University-Joint Institutional Review Board (N201706051).

### 2.2. Data Collection

We retrieved 60,769 subjects who were aged 35 years and above and met MetS criteria from the MJ database between 2004 and 2013. Among 37,392 subjects, after excluding subjects (*n* = 23,377) who had cancer, liver disorder, or renal disease, and 11,376 subjects with missing data, a total of 26,016 subjects were analyzed in this study.

### 2.3. Definition of MetS and Inflammation

The International Diabetes Federation defines individuals with MetS as those who had central obesity based on race- and gender-specific waist circumferences (≥90 cm in men or ≥80 cm in women in Taiwan) and two of the following four factors: (1) elevated systolic blood pressure ≥ 130 mmHg or diastolic blood pressure ≥ 85 mmHg or on treatment for previously diagnosed hypertension; (2) reduced HDL-C < 40 mg/dL (1.03 mmol/L) in men or <50 mg/dL (1.29 mmol/L) in women, or a history of specific treatment for this lipid abnormality; (3) elevated blood serum TG ≥ 150 mg/dL (1.7 mmol/L) or a history of specific treatment for this lipid abnormality; (4) elevated FPG ≥ 100 mg/dL (≥5.60 mmol/L) or previously diagnosed type 2 diabetes mellitus [1]. Additionally, C-reactive protein (CRP) ≥ 28.6 nmol/L was defined as inflammation [29].

### 2.4. Demographic, Lifestyle, and Anthropometric Measurements

The demographic and lifestyle data such as age, gender, education, marital status, smoking, and drinking were obtained using the individual questionnaire, which was conducted when the subjects visited MJ health screening centers before health check-ups [24]. The height, weight, body mass index, and waist circumference were measured during the health check-up as described previously [28].

### 2.5. Blood Pressure and Biochemical Assessment

After overnight fasting for 12–14 h, systolic and diastolic blood pressure were assessed, and blood samples were collected. Total cholesterol, HDL-C, serum TG, FPG, and CRP concentrations were analyzed using commercial reagents [24], whereas low-density lipoprotein-cholesterol (LDL-C) levels were calculated using the formula [30]. All parameters were measured at the central laboratory of the MJ International Health Management Institution.

### 2.6. Dietary Assessment

A validated food frequency questionnaire (FFQ) was used to analyze dietary consumption. The FFQ had 22 food items based on the features of Taiwanese dietary patterns, and each question had five options for the frequency of consumption as servings per day or per week from the lowest to the highest, as described previously [24]. The subjects completed the FFQ before the health check-up at the MJ health screening centers.

### 2.7. Statistical Analysis

Dietary patterns were identified by principal component analysis (PCA). We used orthogonal rotation with the Kaiser criterion (eigenvalues > 1.5) to define dietary patterns [31]. The factor loading of ≥0.30 was used in the classification of dietary patterns. If the food item had a factor loading of ≥0.30 in different dietary patterns, the dietary pattern was identified by which one had a higher factor loading. Factor scores of dietary patterns for each individual summed up the intake of food items weighed by factor loadings, and then classified into tertiles. A chi-square test was used for categorical variables to compare the differences between the characteristics of the subjects and tertiles of dietary pattern scores, while a one-way analysis of variance and the Bonferroni post-hoc test were used to compare continuous variables. A crude or adjusted odds ratio (OR), 95% confidence intervals (CIs), and a multivariable logistic regression model were performed to evaluate the associations of dietary patterns across tertiles of scores with the components of metabolic syndrome and CRP. In the regression model, we categorized the components of MetS and CRP as a high level of mean waist circumference (≥95.8 cm for males and ≥85.2 cm for females), a high level of systolic blood pressure (BP; ≥130 mmHg), a high level of diastolic BP (≥85 mmHg), a low level of HDL-C (<1.03 mmol/L for males and <1.29 mmol/L for females), a high level of serum TG (≥1.70 mmol/L), a high level of FPG (≥5.60 mmol/L), and a high level of CRP (≥28.6 nmol/L). All models were adjusted for age, gender (except waist circumference and HDL-C), education, marital status, smoking, and drinking. The first tertile of dietary pattern scores was considered as a reference. Statistical analyses were conducted using SPSS 24 (IBM Corp., Armonk, NY, USA). Categorical variables are presented as a number (percentage) and continuous variables are presented as a mean ± standard deviation (SD) with *p* values < 0.05 as significant.

## 3. Results

Among 26,016 subjects with MetS, 41.9% and 22.1% had elevated systolic and diastolic blood pressure, respectively, 25.1% had reduced HDL-C, 50.3% had elevated serum TG, 70.1% had abnormal FPG, and 38.8% had elevated CRP (table not shown).

### 3.1. Dietary Patterns

Three dietary patterns identified by PCA are shown in Table 1. The three dietary patterns were derived as the meat–instant food dietary pattern, the vege–seafood dietary pattern, and the cereal–dairy dietary pattern. The three dietary patterns explained 33.45% of total variance. The meat–instant food dietary pattern, the vege–seafood dietary pattern, and the cereal–dairy dietary pattern explained 16.89%, 9.42%, and 7.14% of variance, respectively. The meat–instant food dietary pattern included 10 food items: eggs (i.e., chicken eggs, duck eggs, and quail eggs), meat (i.e., pork, chicken, duck, beef, veal, and lamb), organ meats (i.e., liver, kidneys, and heart), refined dessert (i.e., butter bread, cake, and cookies), sugary drinks, rice/flour cooked in oil (i.e., fried rice and fried noodles), deep-fried food, instant noodle, processed food (i.e., ham, sausage, and canned food), and sauce (i.e., soy sauce, ketchup, hot sauce, and vinegar). The vege–seafood dietary pattern contained six food items: light- or dark-colored vegetables (i.e., carrot, spinach, squash, tomato, cabbage, pechay, cucumber, and radish), fruit, vegetables with oil/dressing, legumes/soy products (i.e., tofu, soybean milk, and dried bean curd), and seafood (i.e., fish, shrimp, and shells). The cereal–dairy dietary pattern included six food items: rice/flour products (i.e., rice, noodle, plain bread, and cruller), whole grains (i.e., whole wheat bread, brown rice, mixed grains, and oatmeal), root crops (i.e., potato, taro, and corn), jam/honey, milk (i.e., fresh milk and powdered milk), and dairy products (i.e., yogurt and cheese).

### 3.2. Characteristics of the Subjects and Dietary Patterns

The characteristics of the subjects across the tertiles of the dietary patterns are summarized in Table 2. Subjects with the higher tertile of the meat–instant food dietary pattern were more likely to be men, younger, current smokers, and drinking at least twice a week, and had adverse components of MetS and higher CRP (*p* < 0.01). On the contrary, subjects with the higher tertile of the vege–seafood dietary pattern or the cereal–dairy dietary pattern had an opposite tendency; they were female, older, not current smokers, and not drinking, and had better components of MetS and lower CRP (*p* < 0.01).

### 3.3. Dietary Patterns, Metabolic Syndrome, and Inflammatory Marker

The odds ratios of the components of MetS and CRP across tertiles of dietary patterns are shown in Table 3, while the association between the components of MetS and CRP are demonstrated in Table S1. The highest tertile (T3) of the meat–instant food dietary pattern was significantly associated with increased crude odds (model 1) of a high level of waist circumference (≥mean of 95.8 cm for male and ≥mean of 85.2 cm for female), a high level of systolic blood pressure (≥130 mmHg), a low level of HDL-C (<1.03 mmol/L for male and <1.29 mmol/L for female), a high level of serum TG (≥1.70 mmol/L), a high level of FPG (≥5.60 mmol/L), and a high level of CRP (≥28.6 nmol/L) compared to the lowest tertile (T1). After adjusting for age, gender, education, marital status, smoking, and drinking (model 2), only a high level of systolic blood pressure (≥130 mmHg) was not significantly correlated with the meat–instant food dietary pattern between the highest tertile and the lowest one.

In contrast, the highest tertile of the vege–seafood dietary pattern was significantly associated with decreased odds of a high level of waist circumference (≥mean of 95.8 cm for male and ≥mean of 85.2 cm for female), a high level of systolic blood pressure (≥130 mmHg), a high level of diastolic blood pressure (≥85 mmHg), a low level of HDL-C (<1.03 mmol/L for male and <1.29 mmol/L for female), a high level of serum TG (≥1.70 mmol/L), a high level of FPG (≥5.60 mmol/L), and a high level of CRP (≥28.6 nmol/L) compared to the lowest tertile in both unadjusted and adjusted models. Similarly, the highest tertile of the cereal–dairy dietary pattern was significantly correlated with decreased odds of a high level of waist circumference (mean of ≥95.8 cm for male and mean of ≥85.2 cm for female), a high level of diastolic blood pressure (≥85 mmHg), a low level of HDL-C (<1.03 mmol/L for male and <1.29 mmol/L for female), a high level of serum TG (≥1.70 mmol/L), a high level of FPG (≥5.60 mmol/L), and a high level of CRP (≥28.6 nmol/L) compared to the lowest tertile in both models.

In Table 3, we did not analyze the association between dietary patterns and body mass index in different genders because we only analyzed the components of MetS and waist circumference as indicators of central obesity, which were included in the components of MetS. The results showed that the association between dietary patterns and waist circumference was statistically significant with similar tendencies in both genders, indicating that there was no gender difference. Moreover, we used the IDF’s definition of MetS, which mentioned that waist circumference was the first criteria to define MetS (male ≥90 cm, female ≥80 cm) in Taiwan; therefore, all subjects had central obesity and we cannot find the odds ratios of waist circumference. Hence, we used mean waist circumference in the table to calculate the odds ratios for both genders.

## 4. Discussion

Our study revealed that the meat–instant food dietary pattern was positively correlated with the components of MetS and CRP among Taiwanese people aged 35 years and above with MetS, while the vege–seafood and cereal–dairy dietary patterns were inversely associated. The meat–instant food dietary pattern was characterized as high in animal protein, saturated fat, sweets, sodium, and food additives. Hence, it was more similar to the Western or unhealthy dietary pattern [24,31]. Consistent with our results, the Western dietary pattern—which is high in saturated fat—was positively correlated with MetS [22,32]. Intake of saturated fat had a positive association with abnormal lipoprotein levels and increased blood lipids and blood pressure [33]. Additionally, high intake of sweets and sugary beverages increased insulin resistance and central obesity [34,35]. The previous study also found that high intake of meat—especially organ meat and processed meat—was strongly correlated with MetS and CVD [36]. Our results suggested that the meat–instant food dietary pattern was more likely to be adopted by younger men, current smokers, or those who were drinking. Similarly, both the Western dietary pattern and unhealthy lifestyles such as smoking and drinking were highly associated with MetS and its components [37,38].

The vege–seafood dietary pattern, which was high in dietary fibers, vitamins, minerals, and unsaturated fat, was comparable to the healthy dietary pattern. The healthy dietary pattern had a protective effect against MetS and its components [39] and CVD [40]. The Mediterranean dietary pattern is associated with decreased risk of MetS. It is rich in fruit, vegetables, fish, and legumes [41]. The components of the healthy dietary pattern such as dietary fiber, vitamins, minerals, mono-, and poly-unsaturated fat may improve insulin sensitivity, increase antioxidative defense, reduce the risk of metabolic disorder, and decrease fatty liver disease [42,43,44].

Comparable with the healthy dietary pattern, the cereal–dairy dietary pattern was characterized as being rich in dietary fiber, antioxidants, phytochemicals, complex carbohydrates, prebiotics, and probiotics. The previous study demonstrated that the whole cereal dietary pattern with high intake of whole grains and whole wheat provided abundant sources of dietary fiber and antioxidants, which was beneficial to health promotion [45]. Previous studies also showed that both whole grains and whole wheat as a prebiotic regulated body weight and insulin action [45,46,47]. Overall, dietary pattern appears to more effectively mediate health problems than individual food items, although some food items had significant effects on health status. Therefore, the inverse association of the vege–seafood or cereal–dairy dietary pattern with the components of MetS and CRP could be attributed to the individual’s overall dietary pattern, rather than single food items.

The meat–instant food dietary pattern had a positive association with CRP, which is a marker of inflammation that can predict CVD [4]. Consistent with our results, previous studies in China and Iran showed that the Western dietary pattern characterized by high intake of red meat, processed meat, butter, high-fat dairy products, sweets, and soft drinks positively correlated with CRP levels [48,49]. Our findings also showed that the vege–seafood or cereal–dairy dietary pattern was inversely associated with CRP levels. They suggested that healthy dietary patterns such as vege–seafood or cereal–dairy dietary pattern had beneficial effects on inflammatory regulation. Similarly, high intake of fruits and vegetables revealed a significantly inverse association with CRP levels [50,51]. Additionally, a randomized controlled clinical trial suggested that probiotics and prebiotics as functional food reduced inflammatory markers such as CRP in patients with type 2 diabetes [52,53]. The prudent pattern—characterized by high consumption of dietary fiber and antioxidants—was also correlated with a decreased level of CRP and risk of CVD [54]. The vege–seafood and cereal–dairy dietary patterns were associated with lower inflammatory marker levels, suggesting that these dietary patterns might reduce the risk of inflammation-related chronic diseases [55].

The components of MetS had significantly greater odds of high CRP levels. Consistently, the subjects with MetS had significantly greater odds of elevated CRP levels compared with those without MetS in nondiabetic Cuban Americans [56]. Central obesity and other components of MetS were correlated with increased CRP levels [56]. The International Diabetes Federation suggested that pro-inflammatory states including rises in levels of high-sensitivity CRP and cytokines, as well as a decrease in plasma adiponectin levels, needed to be added to clinical metabolic parameters of MetS [57]. A suspected mechanism that increased central adiposity and insulin resistance due to chronic low-grade inflammation of MetS might increase the production of CRP in the liver [58]. After adjusting for dietary patterns and other possible confounding variables, our findings report that the odds of elevated CRP levels tended to decrease slightly, but were still significant in all components of MetS, indicating that dietary patterns consistently had an impact on the correlation between the components of MetS and CRP levels. Dietary patterns might influence not only the homeostasis of pro- and anti-inflammatory cytokines or adipokines, but also the state of low-grade inflammation [59].

According to the IDF definition, central obesity is the first step to defining MetS, followed by the other factors. Central obesity was assessed using waist circumference. Additionally, it was associated with insulin resistance and contributed to MetS and its components. Moreover, significant evidence linked larger waist circumferences with the development of CVD [1] and CRP [60]. In Taiwan, central obesity has become an important issue related to metabolic health. The prevalence of central obesity has increased significantly in Taiwanese middle-aged and older adults [13]. Through this study, we expected to show the problems that central obesity and MetS pose to middle-aged and older adults in Taiwan. To our knowledge, this study is the first of its kind to investigate the association of dietary patterns with components of MetS and inflammation among Taiwanese people aged 35 years and above with MetS. Moreover, this cross-sectional study had a large sample size collected from one institution for a period of 10 years, and the data of the participants included demographic information, lifestyle, dietary intake, anthropometric data, and biochemical measurements, which provided a variety of data information and the homogenization of data analysis. However, we had a limitation in this study. We used a simple self-reported FFQ which only provided an estimation of regular dietary intake. In this retrospective observational study, although the questionnaire provided specific frequencies and portions for each food item, we could not collect the intake of a particular food to calculate the intake of a specific nutrient. Additional studies are needed to explore the association of dietary patterns with MetS and inflammation using a longitudinal study.

## 5. Conclusions

Our findings suggest that diet plays an important role in the management of both MetS and inflammation. On one hand, intake of the meat–instant food dietary pattern was positively associated with components of MetS and CRP. On the other hand, intake of the vege–seafood or cereal–dairy dietary patterns was inversely correlated with components of MetS and CRP.

## Figures and Tables

**Table 1:** Factor loadings and dietary patterns derived by principal component analysis for the 22 food groups.

Food Groups	Factor I Meat–Instant Food Dietary Pattern	Factor II Vege–Seafood Dietary Pattern	Factor III Cereal–Dairy Dietary Pattern
Milk	−0.174	0.047	0.597
Dairy products	0.038	0.058	0.634
Eggs	0.471	0.131	0.154
Meat	0.552	0.265	−0.037
Organ meats	0.313	0.127	0.036
Legumes/soy products	0.331	0.354	0.146
Seafood	0.312	0.400	0.070
Light-colored vegetables	−0.055	0.815	0.078
Dark-colored vegetables	−0.084	0.841	0.080
Fruit	−0.063	0.440	0.355
Vegetables with oil/dressing	0.278	0.569	−0.053
Rice/flour products	0.295	−0.011	0.521
Whole grains	−0.039	0.089	0.441
Root crops	0.126	0.241	0.397
Refined dessert	0.327	0.226	−0.079
Jam/honey	0.317	−0.066	0.453
Sugary drinks	0.524	−0.173	0.154
Rice/flour cooked in oil	0.405	0.011	0.238
Deep-fried food	0.674	0.111	−0.030
Instant noodle	0.397	−0.115	−0.009
Processed food	0.611	0.051	0.053
Sauce	0.549	0.076	−0.035

**Table 2:** Characteristics of the subjects across tertiles of dietary patterns (*n* = 26,016) ^1^.

Variables	Meat–Instant Food Dietary Pattern			*p ^2^*	Vege–Seafood Dietary Pattern			*p ^2^*	Cereal–Dairy Dietary Pattern			*p ^2^*
	T1	T2	T3		T1	T2	T3		T1	T2	T3	
*n*	8626	8754	8636		8640	8752	8624		8645	8741	8630	
Factor scores of dietary patterns	−2.67 to −0.5	>−0.5 to 0.27	>0.27 to 5.29		−3.27 to −0.37	>−0.37 to 0.26	>0.26 to 4.79		−2.63 to −0.51	>−0.51 to 0.31	>0.31 to 5.54	
Males	3897 (45.2)	4996 (57.1)	5532 (65.2)	0.000	5141 (59.5)	4790 (54.8)	4594 (53.3)	0.000	5074 (58.7)	5007 (57.3)	4444 (51.5)	0.000
Age (years)	56.3 ± 11.7	54.0 ± 11.4	50.3 ± 11.4	0.000	52.5 ± 11.9	53.5 ± 11.5	54.6 ± 11.8	0.000	52.8 ± 11.5	53.5 ± 11.6	54.2 ± 12.2	0.000
Education ≤High school >High school	4993 (57.9) 3633 (42.1)	4711 (53.8) 4043 (46.2)	3985 (46.1) 4651 (53.9)	0.000	4455 (51.6) 4185 (48.4)	4574 (52.3) 4178 (47.7)	4660 (54.0) 3964 (46.0)	0.004	4967 (57.5) 3678 (42.5)	4443 (50.8) 4298 (49.2)	4279 (49.6) 4351 (50.4)	0.000
Marital status Never married Married Divorced/widowed	199 (2.3) 7048 (81.7) 1379 (16.0)	222 (2.5) 7531 (86.0) 1001 (11.5)	405 (4.7) 7398 (85.7) 833 (9.6)	0.000	358 (4.2) 7184 (83.1) 1098 (12.7)	280 (3.2) 7437 (85.0) 1035 (11.8)	188 (2.2) 7356 (85.3) 1080 (12.5)	0.000	291 (3.4) 7250 (83.8) 1104 (12.8)	247 (2.8) 7495 (85.8) 999 (11.4)	288 (3.3) 7232 (83.8) 1110 (12.9)	0.002
Current smoker No Yes	8333 (96.6) 293 (3.4)	8431 (96.3) 323 (3.7)	8250 (95.5) 379 (4.5)	0.001	8256 (95.6) 384 (4.4)	8416 (96.2) 336 (3.8)	8342 (96.7) 282 (3.3)	0.000	8219 (95.1) 426 (4.9)	8391 (96.0) 350 (4.0)	8404 (97.4) 226 (2.6)	0.000
Drinking <2 times/week ≥2 times/week	8275 (95.9) 351 (4.1)	8373 (95.6) 381 (4.4)	8181 (94.7) 455 (5.3)	0.000	8205 (95.0) 435 (5.0)	8351 (95.4) 401 (4.6)	8273 (95.9) 351 (4.1)	0.01	8152 (94.3) 493 (5.7)	8340 (95.4) 401 (4.6)	8337 (96.6) 293 (3.4)	0.000
BMI (kg/m^2^)	27.1 ± 2.6	27.2 ± 2.6	27.2 ± 2.7	0.000	27.4 ± 2.7	27.1 ± 2.6	27.1 ± 2.7	0.000	27.3 ± 2.7	27.3 ± 2.6	27.0 ± 2.6	0.000
WC (cm)	89.1 ± 6.6	91.1 ± 6.6	93.2 ± 8.6	0.000	92.3 ± 8.6	90.6 ± 6.5	90.5 ± 7.1	0.000	91.9 ± 8	91.7 ± 7.7	89.8 ± 6.2	0.000
Systolic BP (mmHg)	118.5 ± 39.4	120.8 ± 35.2	121.1 ± 33.7	0.000	121.2 ± 35.9	120.6 ± 34.7	118.8 ± 37.9	0.000	122.6 ± 34.7	119.9 ± 36.2	117.8 ± 37.5	0.000
Diastolic BP (mmHg)	70.3 ± 23.5	72.3 ± 21.1	72.4 ± 20.7	0.000	72.4 ± 21.9	71.6 ± 20.8	71.1 ± 22.7	0.001	72.7 ± 20.6	72.1 ± 22.1	70.2 ± 22.6	0.000
TC (mmol/L)	5.2 ± 1	5.3 ± 0.9	5.3 ± 0.9	0.007	5.3 ± 0.9	5.2 ± 0.9	5.2 ± 0.8	0.000	5.3 ± 0.9	5.3 ± 0.9	5.2 ± 0.9	0.000
HDL-C (mmol/L)	1.3 ± 0.4	1.3 ± 0.4	1.2 ± 0.4	0.000	1.2 ± 0.4	1.3 ± 0.4	1.3 ± 0.4	0.000	1.2 ± 0.4	1.3 ± 0.4	1.3 ± 0.4	0.000
LDL-C (mmol/L)	3.6 ± 0.9	3.7 ± 0.8	3.7 ± 0.9	0.000	3.7 ± 0.9	3.6 ± 0.8	3.6 ± 0.8	0.000	3.7 ± 0.9	3.7 ± 0.9	3.6 ± 0.8	0.000
Serum TG (mmol/L)	1.8 ± 1.1	2.0 ± 1.3	2.0 ± 1.5	0.000	2.1 ± 1.3	1.9 ± 1.2	1.9 ± 1.4	0.000	2.1 ± 1.4	1.9 ± 1.3	1.9 ± 1.2	0.000
FPG (mmol/L)	6.1 ± 1.5	6.2 ± 1.6	6.6 ± 2.3	0.000	6.5 ± 2.2	6.3 ± 1.8	6.1 ± 1.5	0.000	6.4 ± 2	6.4 ± 1.9	6.1 ± 1.5	0.000
CRP (nmol/L)	29.7 ± 47.7	32.0 ± 43.3	32.0 ± 45.6	0.001	30.9 ± 45.7	30.4 ± 44.6	28.3 ± 37.4	0.000	31.9 ± 42.3	31.9 ± 41.1	27.7 ± 39.0	0.000

BMI: body mass index, WC: waist circumference, BP: blood pressure, TC: total cholesterol, HDL-C: high-density lipoprotein-cholesterol, LDL-C: low-density lipoprotein-cholesterol, TG: triacylglycerol, FPG: fasting plasma glucose, CRP: C-reactive protein. ^1^ Data are presented as the mean ± SD for continuous variables and *n* (%) for categorical variables. ^2^ *p*-values were derived from general linear regression for continuous variables and from chi-square test for categorical variables.

**Table 3:** Odds ratios (95% confidence intervals) for components of metabolic syndrome ^1^ and C-reactive protein ^2^ across tertiles of dietary patterns.

Components of Metabolic Syndrome		Meat–Instant Food Dietary Pattern		Vege–Seafood Dietary Pattern	Cereal–Dairy Dietary Pattern		
		Model 1 ^3^	Model 2 ^4^	Model 1	Model 2	Model 1	Model 2
High level of WC (male)	T1	1	1	1	1	1	1
	T2	1.293 (1.185–1.412)	1.288 (1.180–1.407)	0.820 (0.756–0.890)	0.827 (0.762–0.897)	0.974 (0.900–1.054)	0.982 (0.907–1.064)
	T3	1.480 (1.360–1.611)	1.479 (1.358–1.610)	0.593 (0.545–0.646)	0.601 (0.552–0.654)	0.777 (0.717–0.843)	0.778 (0.717–0.844)
	*p*	0.000	0.000	0.000	0.000	0.000	0.000
High level of WC (female)	T1	1	1	1	1	1	1
	T2	1.527 (1.400–1.666)	1.512 (1.386–1.649)	0.843 (0.768–0.924)	0.837 (0.763–0.919)	1.003 (0.916–1.099)	0.993 (0.907–1.089)
	T3	1.949 (1.777–2.138)	1.889 (1.720–2.074)	0.816 (0.746–0.892)	0.811 (0.741–0.888)	0.884 (0.808–0.968)	0.882 (0.806–0.996)
	*p*	0.000	0.000	0.006	0.008	0.000	0.000
High level of systolic BP	T1	1	1	1	1	1	1
	T2	1.180 (1.111–1.254)	1.038 (0.973–1.107)	0.912 (0.859–0.969)	0.898 (0.844–0.956)	1.053 (0.991–1.119)	0.988 (0.928–1.051)
	T3	1.240 (1.167–1.318)	1.049 (0.985–1.117)	0.868 (0.817–0.922)	0.843 (0.792–0.897)	1.066 (1.004–1.132)	1.041 (0.979–1.108)
	*p*	0.000	0.337	0.000	0.000	0.088	0.215
High level of diastolic BP	T1	1	1	1	1	1	1
	T2	1.008 (0.937–1.084)	1.020 (0.946–1.099)	0.838 (0.781–0.900)	0.837 (0.779–0.899)	1.071 (0.998–1.149)	1.077 (1.004–1.155)
	T3	1.073 (0.999–1.152)	1.069 (0.995–1.150)	0.817 (0.760–0.877)	0.817 (0.761–0.878)	0.776 (0.721–0.835)	0.788 (0.732–0.849)
	*p*	0.142	0.207	0.000	0.000	0.000	0.000
Low level of HDL-C (male)	T1	1	1	1	1	1	1
	T2	1.370 (1.250–1.501)	1.318 (1.202–1.446)	0.609 (0.559–0.663)	0.620 (0.569–0.676)	0.939 (0.860–1.025)	0.929 (0.851–1.015)
	T3	1.447 (1.318–1.501)	1.453 (1.322–1.597)	0.573 (0.525–0.625)	0.584 (0.535–0.638)	0.883 (0.809–0.964)	0.894 (0.818–0.977)
	*p*	0.000	0.000	0.000	0.000	0.022	0.044
Low level of HDL-C (female)	T1	1	1	1	1	1	1
	T2	1.147 (1.052–1.252)	1.100 (1.007–1.201)	0.823 (0.751–0.903)	0.792 (0.722–0.870)	0.905 (0.826–0.993)	0.864 (0.787–0.949)
	T3	1.515 (1.382–1.661)	1.392 (1.268–1.529)	0.843 (0.770–0.924)	0.833 (0.760–0.914)	0.810 (0.740–0.886)	0.764 (0.697–0.838)
	*P*	0.000	0.000	0.000	0.000	0.000	0.000
High level of serum TG	T1	1	1	1	1	1	1
	T2	1.264 (1.190–1.343)	1.122 (1.054–1.194)	0.803 (0.757–0.853)	0.824 (0.776–0.875)	0.823 (0.776–0.874)	0.835 (0.786–0.886)
	T3	1.356 (1.276–1.440)	1.281 (1.205–1.361)	0.711 (0.670–0.755)	0.742 (0.699–0.789)	0.780 (0.735–0.828)	0.812 (0.764–0.862)
	*p*	0.000	0.000	0.000	0.000	0.000	0.000
High level of FPG	T1	1	1	1	1	1	1
	T2	1.251 (1.172–1.335)	1.318 (1.230–1.412)	0.821 (0.769–0.876)	0.803 (0.751–0.859)	1.079 (1.011–1.152)	1.078 (1.008–1.153)
	T3	1.239 (1.161–1.322)	1.255 (1.173–1.342)	0.885 (0.829–0.945)	0.887 (0.829–0.949)	0.914 (0.857–0.975)	0.929 (0.750–0.857)
	*p*	0.000	0.000	0.000	0.000	0.000	0.000
High level of CRP	T1	1	1	1	1	1	1
	T2	0.891 (0.817–0.973)	0.978 (0.893–1.071)	0.748 (0.691–0.810)	0.761 (0.702–0.825)	1.054 (0.973–1.142)	1.060 (0.978–1.149)
	T3	1.201 (1.106–1.304)	1.257 (1.156–1.367)	0.739 (0.683–0.799)	0.740 (0.699–0.801)	0.965 (0.854–1.062)	0.980 (0.902–1.085)
	*p*	0.000	0.000	0.000	0.000	0.000	0.000

The odds ratios across tertiles of dietary patterns were compared to the reference group (T1). WC: waist circumference, BP: blood pressure, HDL-C: high-density lipoprotein-cholesterol, TG: triacylglycerol, FPG: fasting plasma glucose, CRP: C-reactive protein. ^1^ Components of metabolic syndrome were defined as a high level of WC (mean) (≥95.8 cm for males and ≥85.2 cm for females), a high level of systolic BP (≥130 mmHg), a high level of diastolic BP (≥85 mmHg), a low level of HDL-C (<1.03 mmol/L for males and <1.29 mmol/L for females), a high level of serum TG (≥1.70 mmol/L), a and high level of FPG (≥5.60 mmol/L). ^2^ High level of CRP was defined as ≥28.6 nmol/L. ^3^ Unadjusted. ^4^ Adjusted for age, gender (except WC and HDL-C), education, marital status, smoking, and drinking.

## Supplementary Materials

The following are available online at http://www.mdpi.com/2072-6643/10/2/143/s1. Table S1. Odds ratios (95% confidence intervals) of metabolic syndrome components for a high level of C-reactive protein.
