# Supplementary material for: Association of Dietary Patterns with Components of Metabolic Syndrome and Inflammation among Middle-Aged and Older Adults with Metabolic Syndrome in Taiwan
Source: Nutrients. 2018 Jan 28;10(2):143. doi: 10.3390/nu10020143 (PMC5852719; doi:10.3390/nu10020143)
Supplement: Supplementary file 1 [file nutrients-10-00143-s001.docx]

**Supplementary Material: Association of dietary patterns with components of metabolic syndrome and inflammation among middle-aged and older adults with metabolic syndrome in Taiwan**

Ahmad Syauqy, Chien-Yeh Hsu, Hsiao-Hsien Rau and Jane C-J Chao

|  | **CRP** | |
| --- | --- | --- |
|  | **Model 1 ^3^** | **Model 2 ^4^** |
| WC (male) |  |  |
| < 95.8 cm | 1 | 1 |
| ≥ 95.8 cm | 1.892 (1.733-2.065) ** | 1.775 (1.622-1.942) ** |
| WC (female) |  |  |
| < 85.2 cm | 1 | 1 |
| ≥ 85.2 cm | 1.213 (1.082-1.359) * | 1.172 (1.043-1.316) * |
| Systolic BP |  |  |
| < 130 mmHg | 1 | 1 |
| ≥ 130 mmHg | 1.361 (1.270-1.459) ** | 1.339 (1.246-1.439) ** |
| Diastolic BP |  |  |
| < 85 mmHg | 1 | 1 |
| ≥ 85 mmHg | 1.346 (1.244-1.457) ** | 1.245 (1.148-1.349) ** |
| HDL-C (male) |  |  |
| ≥ 1.03 mmol/l | 1 | 1 |
| < 1.03 mmol/l | 1.537 (1.403-1.683) ** | 1.400 (1.273-1.538) ** |
| HDL-C (female) |  |  |
| ≥ 1.29 mmol/l | 1 | 1 |
| < 1.29 mmol/l | 1.440 (1.285-1.614) ** | 1.411 (1.255-1.586) ** |
| Serum TG |  |  |
| < 1.70 mmol/l | 1 | 1 |
| ≥ 1.70 mmol/l | 1.540 (1.436-1.652) ** | 1.412 (1.315-1.517) ** |
| FPG |  |  |
| < 5.60 mmol/l | 1 | 1 |
| ≥ 5.60 mmol/l | 1.615 (1.488-1.753) ** | 1.499 (1.378-1.632) ** |

**Table S1.** Odds ratios (95% confidence intervals) of metabolic syndrome components ^1^ for high level of C-reactive protein ^2^.

The odds ratios of metabolic syndrome components were compared to the reference group (OR = 1) with lower values using multivariable logistic regression model. WC: waist circumference, BP: blood pressure, HDL-C: high-density lipoprotein-cholesterol, TG: triacylglycerol, FPG: fasting plasma glucose, CRP: C-reactive protein. ^1^ Components of metabolic syndrome were defined as WC (mean): ≥ 95.8 cm for male and ≥ 85.2 cm for female, systolic BP: ≥ 130 mmHg, diastolic BP: ≥ 85 mmHg, HDL-C: < 1.03 mmol/l for male and < 1.29 mmol/l for female, serum TG: ≥ 1.70 mmol/l, and FPG: ≥ 5.60 mmol/l. ^2^ CRP ≥ 28.6 nmol/l. ^3^ Unadjusted. ^4^ Adjusted for dietary patterns, age, gender (except WC and HDL-C), education, marital status, smoking, and drinking. * *p* < 0.05, ** *p* < 0.01.
